# Encouraging job crafting in the workplace for newcomers: A two-year multi-wave study

**DOI:** 10.3389/fpsyg.2022.1003276

**Published:** 2022-12-09

**Authors:** Seoyeong Jeong, Sunyoung Kim, Jeong Hoon Seol, Myongki Lim, Young Woo Sohn

**Affiliations:** ^1^Department of Psychology, Yonsei University, Seoul, South Korea; ^2^Samsung Global Research, Seoul, South Korea

**Keywords:** job crafting, transformational leadership, occupational self-efficacy, calling, multi-wave study

## Abstract

It is important to identify the antecedents of newcomers’ job crafting as it assists with their adjustment in the workplace. This study made use of transformational leadership and newcomers’ calling as organizational and personal resources that predict job crafting. We hypothesized that transformational leadership would have an indirect relationship with newcomers’ job crafting after 2 years through their occupational self-efficacy and that their calling would moderate this mediational path. A multi-wave approach was employed wherein data from 280 new employees were collected three times during the first 2 years of their careers. The survey was completed by 150 participants. The results illustrated that transformational leadership was positively related to newcomers’ job crafting after 2 years of entry through their occupational self-efficacy. Additionally, newcomers’ calling moderated the mediating effect of occupational self-efficacy between transformational leadership and job crafting. The theoretical and practical implications of this study are discussed.

## Introduction

In order to respond to situations with high uncertainty and rapid changes in the organizational or occupational environment, the members of the organization need to be proactive (Griffin et al., 2010). A top-down approach introduced by an organization might be partly effective in addressing the current organizational environment. However, a bottom-up approach initiated by proactive employees remains essential (Wang et al., 2017) as they enhance their performance in a rapidly changing work environment (Petrou et al., 2015). In addition, job crafting, which is one of the proactive behavior, is known to be related to work engagement (Frederick and VanderWeele, 2020), job satisfaction (Slemp et al., 2015; Yeşilkaya and Yıldız, 2022), low burnout (Tims et al., 2013; Pijpker et al., 2022), high performance (Tims et al., 2015; Boehnlein and Baum, 2022), and a person-job fit (Tims et al., 2016), resulting in positive outcomes for both individual and organization. Therefore, investigating factors that could promote the employees’ proactivity is a matter of urgency and this study focuses on the psychological mechanism for encouraging job crafting, which is one of the proactive activities.

The modification of the conditions and boundaries of job tasks, relationships, and the meaning of work is what job crafting entails (Wrzesniewski and Dutton, 2001). It holds significance as it involves a bottom-up approach, which provides the employees with opportunities not only to be productive in the workplace but also for their self-improvement (Tims and Bakker, 2010). Wrzesniewski and Dutton (2001) classified job crafting into three sub-factors: task crafting—instigating the change in job tasks; relational crafting—changing the nature of the interactions at work; and cognitive crafting—modifying the employees’ perspectives on the meaning and importance of the job.

Who has the potential to develop as a job crafter? A model of job crafting presented by Wrzesniewski and Dutton (2001), who first illustrated the overall process in this regard, explains job crafting through the role of work identity, which is developed on the job. In particular, employees’ positive work identity impacts their decisions on their behavior (Golden-Biddle and Rao, 1997), provides self-feedback to employees, and motivates them to continue job crafting (Wrzesniewski and Dutton, 2001). Based on the model of job crafting (Wrzesniewski and Dutton, 2001), many studies have identified the antecedents of job crafting among employees in general (e.g., Rudolph et al., 2017). However, few studies (e.g., Chiesa et al., 2020; Cheng et al., 2022) have focused on job crafting and the proactive behavior among the new graduates or newcomers. Therefore, understanding how to encourage job crafting in an organization among newcomers is somewhat limited.

Investigating antecedents of job crafting among newcomers is extremely important. Newcomers, who leave university and become employees, are in the school-to-work transition phase (Ng and Feldman, 2007; Schoon and Heckhausen, 2019), wherein identities as a student and worker are the most salient (Super, 1990). Further, efficiently identifying a work role is critical in determining school-to-work transition success (Ng and Feldman, 2007; Masdonati et al., 2022). If an organization can help newcomers understand their positive work identity when they establish their attitudes and identities toward their careers, newcomers are more likely to endeavor toward job crafting and immensely benefit from it. As such, this study explores the variables that can predict job crafting among newcomers who become employed immediately after graduating from college.

In particular, this study identified organizational and personal factors as antecedents of job crafting. Some studies have examined the interaction between the leaders’ behavior and newcomers’ personal characteristics in promoting job crafting and enhancing the early organizational adaptation (e.g., Ji and Yoon, 2021; Cheng et al., 2022). Therefore, in line with recent research, this study identified the positive relationship between the transformational leadership and job crafting through occupational self-efficacy. In addition, adopting calling as an individual’s positive occupational attitude, this study examined its effect on the indirect pathway between transformational leadership, occupational self-efficacy, and job crafting.

There are many empirical studies that argue that both the positive organizational factors and the personal psychological resources are needed to form a positive identity (e.g., professional identity, Wang et al., 2016), which is required for an employee’s growth as a job crafter. Firstly, in an organizational context, the role of a positive leader who can promote motivation for job crafting and provide task-based opportunities and psychological support is a critical predisposing factor in promoting job crafting among the employees (Li et al., 2013; Afsar et al., 2019). The proactive identity and attitude of a leader is an important factor in fostering a proactive attitude of subordinates through reciprocal social exchanges (e.g., vicarious learning; Chen et al., 2015), which serves as a key context in establishing one’s identity as a job crafter (Wang et al., 2017). Transformational leadership, in particular, not only motivates members to engage in job crafting but also promotes proactive behaviors by respecting their positive identity, as demonstrated in several previous studies (Afsar et al., 2019; Naeem et al., 2021). Thus, this study examines whether transformational leadership functions as an organizational factor with regard to job crafting.

Secondly, as a personal psychological resource, an individual’s calling can facilitate the forming of one’s identity as a job crafter. A sense of calling as a positive attitude toward work provides a confident response to change and enhances positive engagement in the work environment to achieve goals. This is essential in promoting proactive behavior such as job crafting (Crant, 2000). In addition, the chance to attempt job crafting depends on an individual’s work orientation (Wrzesniewski and Dutton, 2001). In particular, those with a sense of calling are more willing to utilize the support and opportunities from a transformational leader as they consider work to be a key factor in their life (Wrzesniewski et al., 1997). Further, they tend to devote themselves to work (Duffy et al., 2011) and strive to live up to their calling (Duffy and Autin, 2013). The empirical research suggests that providing mentoring and organizational support to individuals with a calling leads to the increased proactive behavior (Cai et al., 2021). This study therefore aimed to empirically explore whether a sense of calling functions as a personal resource that can predict job crafting through its interaction with transformational leadership.

**Figure 1:**
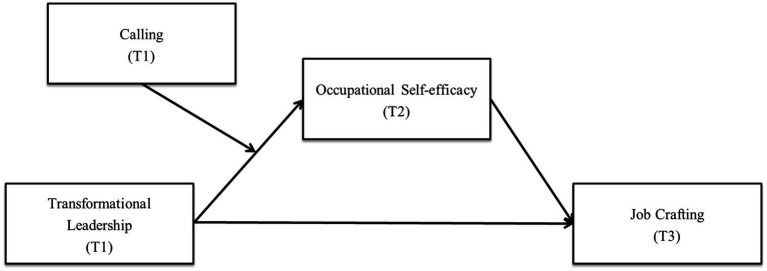
The research model.

Based on the aforementioned call for research, this study identified antecedents that are positively related to job crafting using a multi-wave research design spanned by three different data collection periods over 2 years, starting from the beginning of the newcomers’ careers at their current workplace. The use of this design can minimize common method variance, which is a methodological concern researchers should consider when using self-report surveys (Cooper et al., 2020). This approach theoretically supports the mechanism proposed by Wrzesniewski and Dutton (2001), while providing practical implications for corporate management to assist newcomers with regards to adjusting and nurturing their proactivity. Figure 1 shows this study’s overall model.

## Theoretical background

### Transformational leadership and job crafting

Leaders have tremendous influence on the job crafting behavior as an organizational factor (Zhang and Parker, 2019). There are several recent studies (Wang et al., 2017; Hetland et al., 2018) that have revealed that transformational leaders who provide inspiration toward consistent changes to their subordinates (Bass, 1985; Stock et al., 2022), can promote the employees’ job crafting.

The members of these organizations often define who they are based on the work they do (e.g., role identity theory; Stets and Burke, 2000; Ashforth and Schinoff, 2016; Shepherd and Williams, 2018). Thus, situational and contextual factors considerably influence the establishment of the employees’ identity (Kroger, 1997). Moreover, individuals form their identity through interactions with the various social environments (Bothma et al., 2015). During this process, transformational leaders function as an organizational resource for newcomers to experience growth as proactive crafters based on their self-awareness. In particular, transformational leaders assist employees in understanding their goals and values (Bass, 1985) and enable them to set clear goals and responsibilities (Bass and Stogdill, 1990). Furthermore, transformational leaders act as good role models for newcomers by demonstrating an active response to change and proactiveness (Wang et al., 2016). Moreover, transformational leaders might have a greater influence on newcomers as a result of their need to change their identity from students to workers (Ng and Feldman, 2007; Schoon and Heckhausen, 2019). Consequently, newcomers who received positive influence from their role models undergo growth as proactive members (e.g., work identity) in their new organization and have a greater chance of finding their direction with regards to job crafting and implementing it accordingly (Wrzesniewski and Dutton, 2001).

In addition, transformational leaders might enable their subordinates to try novel ways of working or encourage them to do extra work proactively by setting challenging goals and instilling the confidence to achieve them (Bass, 1985; Stock et al., 2022). The subordinates can attempt to build good relationships with their co-workers as a result of transformational leaders, who tend to be considerate, support each member (Bass, 1985) and endeavor to increase trust as well as cooperation among employees (Carless et al., 2000). The transformational leaders further aid employees in understanding their goals and values (Bass, 1985) and in taking different perspectives of others rather than taking a self-centered view (Stock et al., 2022). Consequently, the subordinates can reflect on the positive impact and contribution of their work to society. Based on the above discussion and the role that a transformational leader actively plays in inducing a positive impact on job crafting among employees, the following hypothesis was established.

*H1*: Transformational leadership has a positive effect on newcomers’ job crafting.

### The mediating effect of occupational self-efficacy

Self-efficacy refers to the trust in one’s ability to achieve a given goal (Bandura, 1997). Specifically, belief in one’s ability to successfully perform work-related tasks is defined as occupational self-efficacy (Rigotti et al., 2008). As newcomers face a new task in their school-to-work transition phase (Ng and Feldman, 2007), examining their self-efficacy may be very important in predicting their workplace behavior and adjustment thereof. Previous research indicates that individual and organizational effectiveness arise in the process of perceived transformational leadership, which increases an employee’s self-efficacy (Nielsen et al., 2009). Based on the previous findings, this study aims to examine the role of the perceived self-efficacy on the relationship between transformational leadership and job crafting among newcomers.

Self-efficacy is formed through the cognitive process of evaluating various experiences (Bandura, 1997). As humans have the tendency to interpret new information based on their prior beliefs about themselves (Schwartz et al., 2011), positive attitudes and thoughts of the self can lead to a positive evaluation of new experiences and information, which encourages self-efficacy (Sung et al., 2015; Kundu and Ghose, 2016). As such, newcomers who have a clear and positive perception of themselves, with the help of a transformational leader, will therefore accept and interpret their experiences with confidence, and thus experience occupational self-efficacy. In addition, transformational leaders demonstrate individualized consideration of their subordinates and encourage intellectual stimulation (Bass and Avolio, 1994; Stock et al., 2022), along with high expectations, confidence that the subordinate can achieve the desired outcomes, and charismatic behaviors involving modeling and exemplary behaviors (Yukl, 1999). The characteristics and behavior of transformational leaders serve as a basis for self-efficacy (Nielsen et al., 2009) through mastery and vicarious experiences provided by the social model and social persuasion (Bandura, 1994).

Previous research suggests that self-efficacy is a predictor of various successful outcomes, such as academic achievement among undergraduate students (Mazzetti et al., 2020) and job crafting among newcomers (Crant, 2000; Parker et al., 2006; Den Hartog and Belschak, 2012). The motivation of humans is formed through the interaction between the desired outcomes and the feasibility (Vroom, 1982). Even if the desired outcomes are established, however, subjective awareness of feasibility is required for the motivation to be strong enough to lead to a practical action. Therefore, self-efficacy, a positive belief that a task is do-able, may act as a predisposing factor for proactive behavior, such as job crafting. The studies on various cultures and occupational groups have indeed revealed that self-efficacy can be a significant predictor of job crafting (Tims et al., 2014; Miraglia et al., 2017; Zhang and Parker, 2019; Wang et al., 2020). In summary, newcomers who have experienced self-efficacy through a transformational leader are likely to be motivated to engage in job crafting based on their positive perceptions of its feasibility. Based on the above discussion, the following hypothesis was established.

*H2*: Occupational self-efficacy mediates the relationship between transformational leadership and job crafting.

### The moderating effect of calling

A calling is one of three types of work orientation (i.e., job, career, calling; Wrzesniewski et al., 1997). The individuals with a sense of calling tend to believe that their job is the most important part of their life and a key factor in defining their identity (Wrzesniewski et al., 1997). The effectiveness of leadership can differ based on situational factors, such as the organizational circumstances and the employee’s characteristics (e.g., Contingency theory; Fiedler, 1967; House, 1971; Hersey and Blanchard, 1984). Further, calling, as one of the subordinates’ characteristics, can interact with organizational factors (Duffy et al., 2018) and enhance positive work attitudes and behaviors. The work orientation specifically includes the calling of acts as a moderating factor influencing job crafting within, as exhibited in Wrzesniewski and Dutton’s (2001) job crafting model. Therefore, this study investigates the role of having a calling, which can interact with transformational leadership.

Transformational leaders encourage employees’ job crafting by challenging the present situation and developing higher goals and values among subordinates (Bass and Stogdill, 1990; Wang et al., 2016). However, people perceive opportunities for job crafting in an organization differently according to their work orientation despite being in the same situation (Wrzesniewski and Dutton, 2001). As a result, the positive relationship between transformational leadership, occupational self-efficacy, and job crafting can differ based on the subordinates’ work orientation despite the transformational leaders providing equal opportunities. The individuals with a job or career orientation may neglect or fail to utilize opportunities for job crafting as they only focus on financial rewards, promotion or success in the workplace. However, people with a calling are more likely to use the opportunities presented to them to craft their job as they consider work significant in their life (Wrzesniewski et al., 1997) and try to pursue their life purpose in the work environment (Dik et al., 2012). Therefore, newcomers with a higher calling tend to actively utilize opportunities provided by transformational leaders.

Further, the subordinates’ need for the support and intervention from their leader can change the effectiveness of the leadership (De Vries et al., 2002). If subordinates require intervention from their leader because they consider it helpful in achieving their work goal, they will accept the leader’s support. Consequently, the effectiveness of the support needed will increase. Newcomers who have a calling might specifically require support from their leader as they need to identify their work role clearly (e.g., school-to-work transition; Ng and Feldman, 2007) and gain greatly value from their work (Wrzesniewski et al., 1997). Therefore, newcomers with a calling are more likely to be affected by transformational leaders than employees with other work orientations. In summation, we expected that newcomers’ high level of calling would increase the positive effect of transformational leadership on occupational self-efficacy. Based on the above, Hypothesis 3 was established accordingly.

*H3a*: Newcomers’ calling moderates the relationship between transformational leadership and occupational self-efficacy. Further, this relationship is stronger when newcomers’ calling is higher (as opposed to when it is lower).

*H3b*: Newcomers’ calling moderates the indirect effect of transformational leadership in promoting job crafting through occupational self-efficacy. Further, this relationship is stronger when newcomers’ calling is higher (as opposed to when it is lower).

## Materials and method

### Participants and procedures

The participants of this study were 280 new employees belonging to a large corporation, which provided a wide range of services from digital devices to financial investment in South Korea. Before collecting the data, we received approval from the university’s institutional review board (IRB). All participants voluntarily participated in this survey, and they had the right to stop answering the questionnaire at any time if they felt uncomfortable.

The questionnaire was administered during three different periods during the first two working years of new employees. To recruit the participants, online survey links were sent through e-mail. At the end of the questionnaire at Time 1, the respondents were asked whether they wanted to participate in two more surveys; only the respondents who agreed to further participation in the study provided their e-mail addresses. Subsequent survey links at Times 2 and 3 were sent through e-mail. This data collection process was done to minimize the common method variance and increase the validity of this study, even though this long-term process might become a burden for the participants,

In the first wave (T1), 280 participants responded to measures of perceived transformational leadership, their calling, and their demographic questionnaire, such as their age, gender, and religion status during their first week of newcomer orientation. Those who agreed to further participate voluntarily provided their e-mail address. In the second wave (T2), one year later, 187 questionnaires were collected for occupational self-efficacy and the response rate was 66.7%. In the third wave (T3), 1 year later (i.e., 2 years after T1), 150 respondents answered the questionnaires with regard to job crafting, and the final response rate was 53.2%.

After the data collection, the responses were matched using the e-mail addresses of the participants. The participants were given a unique random ID to manage their data, guarantee their anonymity, and eliminate their e-mail addresses from the data for analysis. The systematic attrition of participants from the study was a methodological concern, the independent *t*-tests were therefore performed and compared participants who answered the entire questionnaire to those who dropped out of the study. To assess the attrition, the T1 data for the multi-wave sample (*n* = 150) from the participants who completed the study and the T1 data from participants who dropped out at T2 or T3 (*n* = 130) were compared using a *t*-test. This analysis revealed no significant differences between the multi-wave and attrition groups in terms of age [*t*(278) = 0.29, *p* > 0.05], gender [*t*(278) = −0.76, *p* > 0.05], religious status [*t*(278) = 0.70, *p* > 0.05], transformational leadership [*t*(278) = −1.03, *p* > 0.05], and calling [*t*(278) = 0.62, *p* > 0.05]. These results suggest that attrition was not systematic concerning the study variables.

Finally, data from the 150 respondents who participated in all three questionnaire surveys were used in analyzing the data. The final participants were all South Korean, primarily men (72.7%, *n* = 109), had a religion (87.3%, *n* = 131), and at least four-year university degrees (91.3%, *n* = 137). The participants’ ages ranged from 21 to 30 years, with an average age of 25.10 (*SD* = 1.75).

### Instruments

To measure the calling, the occupational self-efficacy, and job crafting at T1, T2, and T3, respectively, validated the Korean versions of questionnaires which were administered to the participants. The Global Transformational Leadership Scale (GTL) was not validated in South Korea, the authors therefore, carefully translated the items using the translation and back-translation method (Hui and Triandis, 1985; Cha et al., 2007).

#### Transformational leadership

The transformational leadership perceived by the employee was measured at T1 using the Global Transformational Leadership Scale (GTL) developed by Carless et al. (2000). This scale consists of seven sub-dimensions: vision, staff development, support, empowerment, innovative thinking, leading by example, and charisma. The sample items for vision and staff development included “My leader communicates a clear and positive vision of the future” and “My leader treats staff as individuals, supports and encourages their development,” respectively. The followers responded to 7-items on a 5-point Likert scale ranging from 1 (*not at all*) to 5 (*very likely*). The higher the total score of the GTL, the higher the perception of a leader’s transformational leadership. The Cronbach’s alpha was.91 in this study.

#### Calling

The calling an employee had was measured at T1, using the Korean Version of the Calling and Vocation Questionnaire (CVQ-K; Shim and Yoo, 2012), based on the CVQ developed by Dik et al. (2012). In this study, among 24 total items, 12 items measuring the presence of the calling were used. This twelve-item inventory consists of three sub-dimensions: the transcendent summons (e.g., I believe that I have been called to my current line of work), the purposeful work (e.g., My work helps me live out my life’s purpose), and the prosocial orientation (e.g., The most important aspect of my career is its role in helping to meet the needs of others), with each dimension composed of four items. Each item was rated on a 5-point Likert scale ranging from 1 (*not at all true of me*) to 5 (*absolutely true of me*). The higher the total score on the CVQ-K, the higher the perception of the occupational calling. The Cronbach’s alpha was.88 in this study.

#### Occupational self-efficacy

The occupational self-efficacy was assessed at T2, using the 8-item scale developed by Jones (1986) and translated by An (2009), to assess work-related self-efficacy. In An (2009)’s study, the exploratory and confirmatory factor analysis was conducted to confirm the factor structures and revealed the positive relationship between transformational leadership and occupational self-efficacy. This measurement also has been widely used in studies on various employee groups, such as newcomers, teachers, counselors, and salespersons to test the relationship between the occupational self-efficacy and work-related outcomes (Park et al., 2016). Each item was rated on a 5-point Likert scale ranging from 1 (*strongly disagree*) to 7 (*strongly agree*). The examples included “My new job is well within the scope of my abilities” and “I do not anticipate any problems in adjusting to work in this organization.” The higher the total score on this scale, the higher the perception of occupational self-efficacy. The reliability of this scale (Cronbach’s alpha) was.85 in this study.

#### Job crafting

The job crafting was measured at T3, using the Korean version of the Job Crafting Questionnaire (JCQ-K), which was validated by Lim et al. (2014) based on the original JCQ developed by Slemp and Vella-Brodrick (2013). This 15-item inventory consists of three sub-dimensions: task crafting (e.g., Introduce new approaches to improve your work), cognitive crafting (e.g., Think about how your job gives your life purpose), and relational crafting (e.g., Make an effort to get to know people well at work). Each item was rated on a 6-point Likert scale ranging from 1 (*strongly disagree*) to 6 (*strongly agree*). The higher the total score of the JCQ, the higher the level with regard to job crafting. The reliability of this scale (Cronbach’s alpha) was 0.92 in this present study.

Even though job crafting is classified into three sub-factors, we investigate job crafting as one construct rather than considering the three dimensions separately. However, it is possible that there are differences among the three sub-dimensions related to job crafting. We have therefore conducted further data analysis to confirm this when the three dimensions are examined separately. However, the results were not different and we provided them in the Appendix. We therefore used one overall variable in relation to job crafting for further analysis.

### Statistical analysis

We conducted the preliminary analyzes and descriptive statistics using the SPSS 25.0, to examine the skewness and kurtosis of the data regarding the research variables. Furthermore, this study was a three-wave multi-wave research design, it was therefore necessary to ensure that the samples were systematically reduced. Therefore, we performed an independent *t*-test comparison between the samples in which the participants responded at all time points, and the samples in which the participants did not respond at all time points, resulting in the samples being disregarded.

We used Mplus 6.0 to evaluate this study’s measurement model to confirm that the four study variables (e.g., transformational leadership, calling, occupational self-efficacy, and job crafting) could be distinguished from each other. We examined this study’s model using the following model fit indices based on the Hu and Bentler’s (1999) guidelines: the chi-square ($\chi^{2}$), the comparative fit index (CFI), the Tucker-Lewis index (TLI), the standardized root-mean-square residual (SRMR), and the root-mean-square error of approximation (RMSEA).

This study was designed to confirm a moderated mediation model. The moderated mediation means that the mediation effect changed depending on the level of the moderator (Muller et al., 2005). In this study, we hypothesized a mediating relationship between the transformational leadership, the occupational self-efficacy, and the job crafting, as well as the moderating effect of having a calling on the mediation path. Although it was recommended to use the structural equation modeling (SEM) to analyze the hypothesis models, the relatively small sample size due to the multi-wave study design contributed to the lack of power for using the structural equation models (Westland, 2010). For this reason, we used the SPSS PROCESS Macro to test the hypotheses and examine the conditional indirect effects suggested by Hayes (2013).

## Results

### Preliminary analysis

We examined the skewness and kurtosis of the data for the research variables using the guidelines of Weston and Gore (2006). The skewness and kurtosis value of the study variables were less than what was set to be the recommended thresholds (skewness > |3|or kurtosis >|10|).

### Descriptive statistics and correlation analysis

Table 1 displays the results of the descriptive statistics as well as the correlation coefficients of the study variables. This study’s analysis revealed that all our study variables were significantly correlated with each other. Specifically, the transformational leadership was positively related to having a calling (*r* = 0.33, *p* < 0.01), the occupational self-efficacy (*r* = 0.31, *p* < 0.01), and job crafting (*r* = 0.35, *p* < 0.01). The calling was also positively related to occupational self-efficacy (*r* = 0.33, *p* < 0.01) and job crafting (*r* = 0.55, *p* < 0.01). Lastly, a positive relationship was found between the occupational self-efficacy and job crafting (*r* = 0.36, *p* < 0.01). However, the demographic variables such as gender, age, education level, and religious status were not significantly correlated with the main variables of this study.

**Table 1:** The descriptive statistics and correlation analysis.

	*M*	SD	1	2	3	4
1. Transformational leadership (T1)	3.81	0.68	–			
2. Calling (T1)	3.26	0.63	0.33	–		
3. Occupational self-efficacy (T2)	4.69	0.88	0.31	0.33	–	
4. Job crafting (T3)	4.24	0.72	0.35	0.55	0.36	–

n = 150, All correlations were statistically significant (*p* < 0.01).

### Confirmatory factor analysis

To examine the discrimination of the measured constructs, we conducted a confirmatory factor analysis using Mplus 6.0. The results showed that the four-factor model (i.e., T1 transformational leadership, T1 calling, T2 occupational self-efficacy, and T3 job crafting) demonstrated a good fit to this study’s data, $\chi^{2}$(48) = 87.14, *p* < 0.001, CFI = 0.95, TLI = 0.94, RMSEA = 0.07 [90% confidence interval = 0.04 to 0.09], SRMR = 0.05. The results indicated that no other models showed a better fit than the four-factor model, as shown in Table 2.

**Table 2:** The confirmatory factor analysis results.

Category	$\chi^{2}$	df	$\chi^{2}/df$	CFI	TLI	RMSEA	SRMR
Model 1 (1 factor)	500.28	54	9.26	0.52	0.42	0.23	0.14
Model 2 (2 factors)	372.20	53	7.02	0.66	0.58	0.20	0.18
Model 3 (3 factors)	239.26	51	4.69	0.80	0.74	0.15	0.14
Research Model (4 factors)	87.14	48	1.81	0.95	0.94	0.07	0.05

*n* = 150, CFI, Comparative fit index; TLI, Tucker-Lewis index; RMSEA, Root-mean square error of approximation; SRMR, Standardized root-mean square residual. Model 1 (1 factor): All items are loaded on one factor. Model 2 (2 factors): T1 (Transformational leadership, Calling), T2/3 (Occupational self-efficacy, Job crafting). Model 3 (3 factors): T1 (Transformational leadership, Calling), T2 (Occupational self-efficacy), T3 (Job crafting). Research Model (4 factors): Transformational leadership, Calling, Occupational self-efficacy, Job crafting.

### Tests of hypotheses

We expected that calling would moderate the indirect relationship between transformational leadership, occupational self-efficacy, and job crafting. To examine this moderated mediation model, we used the approach suggested by Muller et al. (2005), according to whom there were three statistical stages: the moderation analysis, the mediation analysis, and the moderated mediation analysis, as shown in Table 3.

**Table 3:** The results for testing the hypotheses.

	β	SE	t	LLCI	ULCI	$R^{2}$	F
**Step 1: Moderation analysis (Moderation effect of calling between Transformational leadership and Job crafting)**							
**Outcome variable: Job crafting**							
Constant	–	0.05	84.87^***^	0.40	0.72	0.58	25.55^***^
Transformational leadership	0.20	0.07	2.67^**^	0.05	0.34		
Calling	0.25	0.08	6.96^**^	0.40	0.72		
Transformational leadership × Calling	−0.02	0.08	−0.25	−0.19	0.15		
**Step 2: Mediation analysis**							
**Outcome variable: Occupational self-efficacy**							
Constant	3.15	0.38	8.10^***^	2.38	3.91	0.31	16.37^***^
Transformational leadership	0.40	0.10	4.04^***^	0.20	0.60		
**Outcome variable: Job crafting**							
Constant	2.08	0.36	5.72^***^	1.36	2.79	0.44	18.12^***^
Transformational leadership	0.28	0.08	3.43^***^	0.11	0.44		
Occupational self-efficacy	0.23	0.06	3.61^***^	0.10	0.35		
**Step 3: Moderated mediation analysis**							
**Outcome variable: Occupational self-efficacy**							
Constant	4.66	0.06	68.33^***^	4.52	4.79	0.42	11.00^***^
Transformational leadership	0.29	0.10	2.91^**^	0.09	0.49		
Calling	0.35	0.11	3.17^**^	0.13	0.57		
Transformational leadership × Calling	0.25	0.12	2.11^**^	0.01	0.49		
**Outcome variable: Job crafting**							
Constant	3.15	0.30	10.34^***^	2.55	3.75	0.44	18.12^***^
Transformational leadership	0.25	0.08	3.43^***^	0.11	0.44		
Occupational self-efficacy	0.23	0.06	3.61^***^	0.10	0.35		

n = 150, Bootstrap sample size = 10,000. LLCI, Lower Limit Confidence Interval; ULCI, Upper Limit Confidence Interval. ^*^p < 0.05, ^**^p < 0.01, ^***^p < 0.001.

Firstly, we tested the total direct effect of transformational leadership on job crafting, and the results showed that the transformational leadership was significantly and positively related to job crafting (*β* = 0.20, *SE* = 0.07, *p* < 0.01); thus, Hypothesis 1 was supported. In stage 1, we confirmed a statistically insignificant interaction effect of calling on the relationship between the transformational leadership and job crafting (*β* = −0.02, *SE* = 0.08, *p* = 0.79). These results indicated that the amount of the total direct effect did not change depending on the level of calling (Muller et al., 2005).

In the next stage, we examined the mediation model of transformational leadership, occupational self-efficacy, and job crafting without the moderator by using the multiple regression analysis and Hayes’s (2013) SPSS Macro PROCESS Macro Model 4. The results showed that transformational leadership was significantly related to occupational self-efficacy (*β* = 0.40, *SE* = 0.10, *p* < 0.001). Transformational leadership was also significantly related to job crafting (*β* = 0.28, *SE* = 0.08, *p* < 0.001) even after controlling for occupational self-efficacy (*β* = 0.23, *SE* = 0.06, *p* < 0.001). We also tested the statistical significance of the indirect effect using the bootstrapping analysis (Shrout and Bolger, 2002). The bias-corrected 95% bootstrapping, the confidence interval did not include zero [BC 95% CI (0.03, 0.17)]. These results indicate that the indirect effect is statistically significant. Overall, Hypothesis 2 was supported.

**Figure 2:**
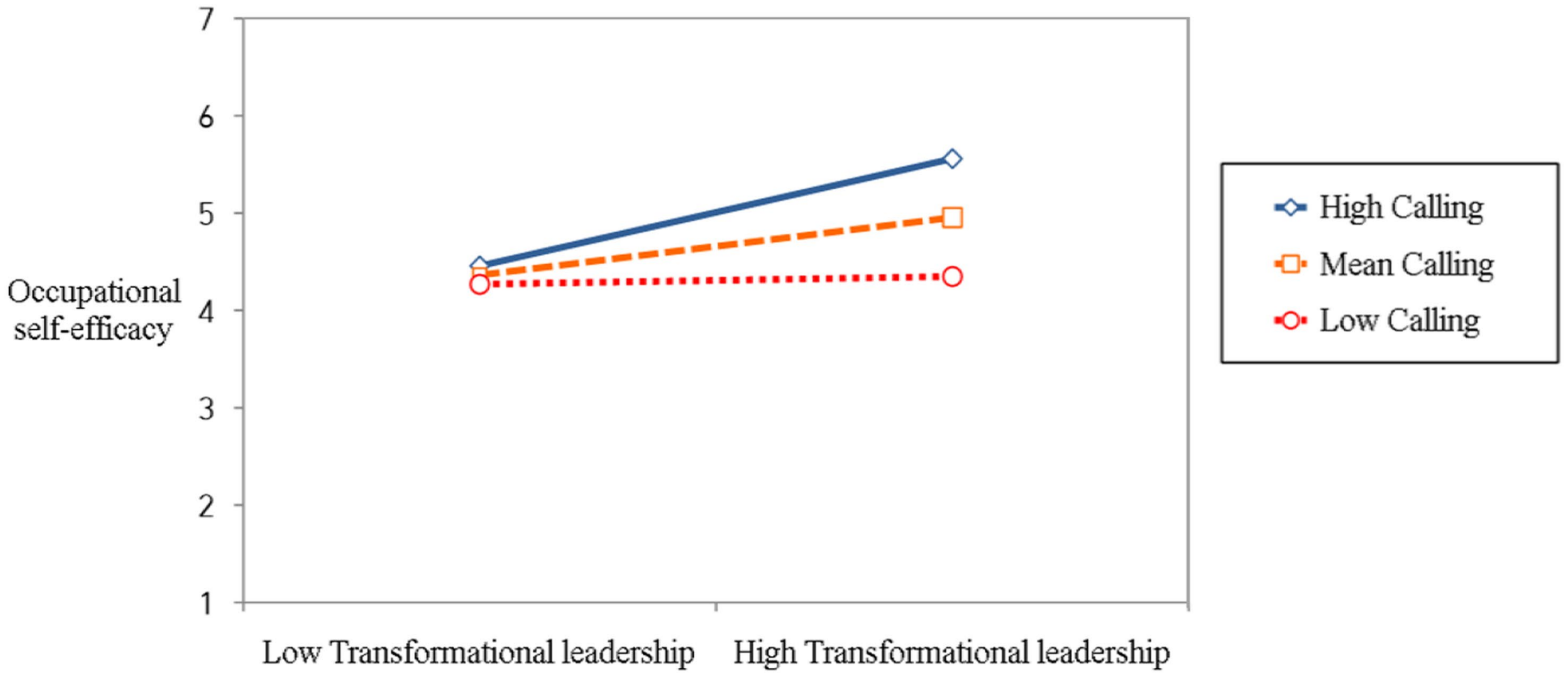
The interaction between transformational leadership and the calling on occupational self-efficacy.

Finally, we tested the moderated mediation model using the SPSS PROCESS Macro Model 7. Hypothesis 3 proposed that the indirect relationship between transformational leadership and job crafting through the occupational self-efficacy was stronger for those who had a calling. We examined whether the indirect effect of mediational relationships changed depending on the level of calling. The interaction term (transformational leadership × calling) was significantly associated with the occupational self-efficacy (*β* = 0.25, *SE* = 0.12, *p* < 0.05), indicating that the effect of the transformational leadership on the occupational self-efficacy varied with the level of calling, as shown in Figure 2. These results supported Hypothesis 3-a.

**Figure 3:**
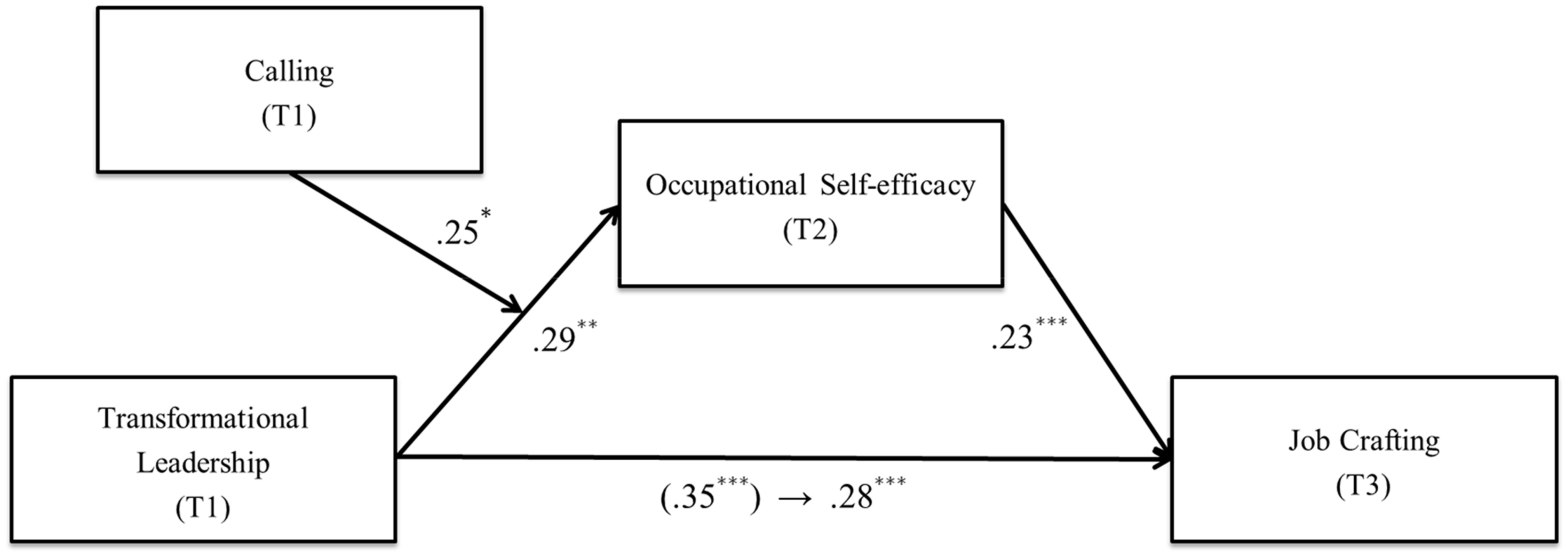
The moderated mediation model with the standardized coefficients. The number in parentheses is the direct path coefficient before the other variables are entered. ^*^p < 0.05, ^**^p < 0.01, ^***^p < 0.001.

To confirm Hypothesis 3-b, we calculated the index of moderated mediation (Hayes, 2015). As shown in Table 4, the results revealed that the index was statistically significant [index = 0.05, *SE* = 0.03, BC 95% CI (0.04, 0.20)], indicating that the indirect effect of transformational leadership on job crafting was moderated by calling. We additionally examined the conditional indirect effects on the values of the moderator (the mean, one standard deviation above, one standard deviation below), as shown in Table 5. The indirect effect was statistically significant for both the high calling (+1*SD*) [*standardized indirect effect* = 0.10, 95% CI (0.04, 0.20)], and mean [*standardized indirect effect* = 0.06, 95% CI (0.02, 0.14)]. However, in the circumstance of an employee having a low calling (−1*SD*), the indirect effect was not significant [*standardized indirect effect* = 0.03, 95% CI (−0.02, 0.10)]. These results suggest that the indirect effect of transformational leadership on job crafting through occupational self-efficacy could be achieved under certain conditions of high (+1*SD*) and mean calling of newcomers, which might not be achieved under a low calling (−1*SD*). Therefore, Hypothesis 3-b was supported (Figure 3).

**Table 4:** The index of moderated mediation.

	Index	Boot SE	95% boot LLCI	95% boot ULCI
Calling	0.06	0.03	0.01	0.14

Bootstrap sample size = 10,000, 95% Boot LLCI = 95% Bootstrapping Lower Limit Confidence Interval, 95% Boot ULCI = 95% Bootstrapping Upper Limit Confidence Interval.

**Table 5:** The results for the conditional indirect effect analysis.

Calling	Indirect effect	Boot SE	95% boot LLCI	95% boot ULCI
+1SD (+0.63)	0.10	0.04	0.04	0.20
Mean	0.06	0.02	0.02	0.14
−1SD (−0.63)	0.03	0.03	−0.02	0.10

Bootstrap sample size = 10,000, 95% Boot LLCI = 95% Bootstrapping Lower Limit Confidence Interval, 95% Boot ULCI = 95% Bootstrapping Upper Limit Confidence Interval.

## Discussion

Job crafting is an essential employee behavior from the perspective of organizational prosperity. For newcomers, job crafting is especially important as it can be a strong indicator of successful adjustment to the workplace (Crant, 2000; Saks et al., 2011). This two-year multi-wave study on newcomers aimed to identify the antecedents that predict job crafting as well as its mechanism. We examined organizational and personal factors based on the role of work identity that influences job crafting attempts, as suggested by Wrzesniewski and Dutton (2001). The transformational leadership was selected as an organizational factor aiding the formation of a positive identity, which enables newcomers to experience growth as a job crafter. The calling was selected as a personal psychological resource. This study specifically examined whether the transformational leadership perceived by newcomers at the beginning of their careers (T1) was positively correlated to job crafting after 2 years (T3), mediated by their occupational self-efficacy 1 year after starting the job (T2). Furthermore, this study aimed to explore whether newcomers’ sense of calling moderates the mediating effect of the relationship between the transformational leadership and occupational self-efficacy on newcomers’ engagement in job crafting.

The main findings are as follows. Firstly, the transformational leadership perceived by newcomers in T1 showed a positive association with job crafting in T3. Additionally, this was mediated by the occupational self-efficacy experienced in T2. This suggests that transformational leadership was not only able to predict job crafting directly but was also indirectly related to job crafting through the occupational self-efficacy. Secondly, the findings confirmed that having a calling moderated the relationship between transformational leadership in T1 and occupational self-efficacy experienced in T2. Newcomers with a relatively high sense of calling (+1*SD*, mean level) felt a greater sense of occupational self-efficacy when connected to a transformational leader compared to those with a relatively low sense of calling (−1*SD*). Thirdly, a moderated mediation analysis demonstrated that the calling moderated the relationship between transformational leadership and job crafting through occupational self-efficacy. That is, a significant conditional indirect effect of calling on the mediation path was observed in newcomers, especially, with an average or above-average level of calling (+1*SD*). Thus, this indicates that for newcomers to grow as proactive job crafters, their calling plays an important role in receiving support from transformational leaders as one of the organizational resources.

### Theoretical implications

This study explored the organizational and personal factors which are positively related to job crafting based on the job crafting model proposed by Wrzesniewski and Dutton (2001). Focusing on how work identity affects job crafting, transformational leadership and calling were positively related to job crafting by promoting the development of positive identities. A leader’s role has been identified as an essential factor in promoting job crafting (Zhang and Parker, 2019). Previous research revealed the positive impact of transformational leadership on employees’ proactive job crafting (Wang et al., 2017; Hetland et al., 2018). The findings of this study not only offer theoretical support for leaders’ positive influence on employees but also provide empirical evidence to the positive influence of transformational leadership. This study also revealed that newcomers’ sense of calling functions as an individual resource, supporting the establishment of a clear identity as a job crafter. These results not only serve as empirical evidence for work orientation, another core individual factor proposed in the job crafting model (Wrzesniewski and Dutton, 2001), but also supports previous research findings that demonstrated a positive work attitude (Bunderson and Thompson, 2009), and motivation in individuals with a sense of calling (Duffy and Autin, 2013). Furthermore, since newcomers start forming their occupational identity in a workplace for the first time, calling can have an even more significant impact on the construction of proactive attitudes.

This study also demonstrates the specific mechanisms underlying these variables. Previous research has demonstrated that self-efficacy mediates transformational leadership and positive individual and organizational outcomes perceived by the employees (Nielsen et al., 2009). This study revealed that occupational self-efficacy, the belief that one can successfully perform a task, is a mediating factor promoting proactive behavior among newcomers. This is consistent with previous research suggesting that self-efficacy is an important factor in promoting proactivity among newcomers experiencing the workplace environment for the first time (Crant, 2000).

Furthermore, this study identified the role of transformational leadership as an organizational resource and role of calling as an individual resource. Through these findings and previous research (e.g., A job crafting model; Wrzesniewski and Dutton, 2001), we can clearly articulate an integrated mechanism that explains why and how newcomers engage in job crafting. More specifically, newcomers in their school-to-work transition period are required to establish their identity as a worker (Ng and Feldman, 2007). The transformational leaders have a positive effect on their job crafting by clearly explaining goals and values. In addition, their calling also helps newcomers become aware of their identity as a worker because it contributes to their definition of themselves.

This study also identified the role of a calling as a moderating factor, reaffirming the interaction between the organizational factors and individual characteristics. In order to verify the effectiveness of leadership, it is necessary to observe not only the leader’s behavior, but also the situational factors, such as the circumstances of the organization and the subordinates’ characteristics (e.g., Contingency theory; Fiedler, 1967; House, 1971; Hersey and Blanchard, 1984). Based on the above suggestion, this study took an integrated approach to explore the mechanism by which transformational leadership encourages job crafting in addition to the moderating function of a calling, an employee characteristic. Furthermore, this study supported the Work as Calling Theory (WCT; Duffy et al., 2018), suggesting that a calling interacted with organizational support and led to various outcomes. The significance of this study lies in the fact that it provides empirical evidence for yet another positive finding (e.g., occupational self-efficacy, job crafting) of the interaction between a calling and organizational support, as suggested in the WCT model.

Lastly, this study minimizes the common method variance by adopting the multi-wave research design. The research topics in the vocational behavior field are often personal and subjective, making the self-report surveys ideal tools for measuring the subjective constructs (Conway and Lance, 2010). However, significant methodological concerns, such as a common method variance, existed in regard to the cross-sectional self-report data from the same respondent. The common method variance can decrease the validity and accuracy of the results of a study because it causes artificial inflation of the parameter estimates (Podsakoff et al., 2012) while deflating the effects on the parameter estimates specifically examining the interaction effects (Siemsen et al., 2010). To address such a significant problem, temporal separation was used during data collection thus contributing to this field of study.

### Practical contribution

This study provides insight into how newcomers can develop into proactive crafters, as well as useful guidance to practitioners managing newcomers in real corporate and organizational environments. Firstly, the findings of this study suggest methods to increase the job crafting among newcomers in organizations. It will be effective to support transformational leaders in educating newcomers and improving their occupational self-efficacy as well as providing transformational leadership training (Kelloway et al., 2000) to managers working with newcomers to encourage job crafting. Many companies have recognized the necessity of job crafting and have utilized intervention programs to promote it (van den Heuvel et al., 2015; Sakuraya et al., 2016; van Wingerden et al., 2017; Verelst et al., 2021), thus the results of this study can significantly contribute to the field. However, corporate management or HR team should carefully interpret and utilize the results of this study as this study cannot confirm the causal relationship between the different variables.

It will also be necessary to support newcomers in discovering their sense of calling as well as engaging in work with a positive attitude and demonstrating proactiveness. People with positive work orientation such as calling can fully utilize these opportunities to craft their work. Employees who experience a sense of calling gain confidence in themselves based on various experiences of success and can have self-efficacy (Hall and Chandler, 2005). Also, people with a calling have high intrinsic motivation and proactive attitude, so these positive characteristics of calling can encourage job crafting (Chang et al., 2020). As such, education (Harzer and Ruch, 2016) and counseling (Dik et al., 2009) could support newcomers in understanding and finding their calling clearly. Since research suggests that newcomers’ calling is reduced during the transition phase from school-to-work (Zhang et al., 2021), identifying strategies to maintain and promote newcomers’ calling is important.

Finally, it is necessary for newcomers to experience occupational self-efficacy, which was found to mediate the relationship between transformational leadership and job crafting. As such, newcomers should be provided with opportunities and interventions to cultivate self-efficacy in a new workplace environment. Empirical research (e.g., van den Heuvel et al., 2015; van Wingerden et al., 2017; Costantini et al., 2022) has further revealed that providing intervention programs can enhance self-efficacy.

### Limitations

Despite numerous theoretical and practical implications, this study has several limitations. Firstly, this study focused on newcomers in Korean corporations who are part of the East Asian culture and have a relatively high level of education. As such, it may be difficult to generalize or replicate the study findings when exploring a different sample. Thus, additional follow-up studies on newcomers in various cultures and organizational environments are needed.

Secondly, all variables in this study were measured using self-reported surveys. Although self-reported measurement is a good method of subjectively perceived leadership and individual status, there is potential for social desirability bias, which, in turn, can lead to difficulties in accurate measurements (Del Boca and Noll, 2000) and common method bias, which is prevalent in studies that rely on self-report measures (Cooper et al., 2020). Given that this study aimed to examine the existence of a positive relationship between newcomers’ perceived transformational leadership and their calling through occupational self-efficacy, a self-report survey was inevitably adopted. However, the common method variance was minimized by employing a multi-wave research design. Nevertheless, future research should increase the validity of the study’s findings by incorporating various methods including more data that is objective, such as the newcomers’ initial onboarding data or evaluations from leaders or colleagues.

Finally, due to the limitations of the multi-wave research design, the causality between study variables cannot be confirmed precisely. To establish a causal relationship between variables, researchers should measure the study variables at least three times by utilizing repeated measure to identify intra-individual change with strong evidence of a causal relationship (Taris et al., 2021). Multi-wave data collection involving only temporal separation without repeated measures is insufficient to confirm a causal relationship. Indeed, some research suggests that job crafting can positively relate to the leader–member exchange (Wang et al., 2018), subsequently affecting the leader in the long term. Many studies have thus emphasized the need for longitudinal research to clarify causal or reciprocal relationships (Hetland et al., 2018; Tims et al., 2021). Further, some empirical studies have postulated the effectiveness of self-efficacy in promoting proactive behavior among undergraduate students before they start employment (e.g., Mazzetti et al., 2020). Therefore, people who perceive high self-efficacy may attempt job crafting without support from an organization. Future research should therefore investigate the causal relationship between the study variables using a longitudinal research approach.

Despite such limitations, this 2-year multi-wave study is important because it expands the predisposing factors of job crafting into leadership and calling. Furthermore, the findings of this study will provide support for the establishment of intervention plans to help newcomers adjust to the early stages of joining a company to increase their proactiveness.

## Data availability statement

The raw data supporting the conclusions of this article will be made available by the authors, without undue reservation.

## Ethics statement

The studies involving human participants were reviewed and approved by the Yonsei University Institutional Review Board. The patients/participants provided their written informed consent to participate in this study.

## Author contributions

ML recruited the participants and collected the data. SJ wrote and led the drafting of the manuscript as a first author. JS analyzed the research model with SJ. JS and SK reviewed the draft and provided essential feedback. YS supervised the whole process of this study and revised the manuscript as a corresponding author. All authors contributed to the article and approved the submitted version.

## Conflict of interest

Author ML was employed by Samsung Global Research.

The remaining authors declare that the research was conducted in the absence of any commercial or financial relationships that could be construed as a potential conflict of interest.

## Publisher’s note

All claims expressed in this article are solely those of the authors and do not necessarily represent those of their affiliated organizations, or those of the publisher, the editors and the reviewers. Any product that may be evaluated in this article, or claim that may be made by its manufacturer, is not guaranteed or endorsed by the publisher.

## Supplementary material

The Supplementary material for this article can be found online at: https://www.frontiersin.org/articles/10.3389/fpsyg.2022.1003276/full#supplementary-material

## References

[ref1] Afsar B. Masood M. Umrani W. A. (2019). The role of job crafting and knowledge sharing on the effect of transformational leadership on innovative work behavior. Pers. Rev. 48, 1186–1208. doi: 10.1108/PR-04-2018-0133

[ref2] An S. (2009). *A study on the mediating effect of self-efficacy and job-involvement on the relationship between transformational leadership and job performance*. doctoral dissertation. Seoul: Hanyang University.

[ref3] Ashforth B. E. Schinoff B. S. (2016). Identity under construction: how individuals come to define themselves in organizations. Annu. Rev. Organ. Psych. Organ. Behav. 3, 111–137. doi: 10.1146/annurev-orgpsych-041015-062322

[ref4] Bandura A. (1994). “Self-efficacy” in Encyclopedia of Human Behavior. ed. Ramachaudran V. S., vol. 4 (New York: Academic Press), 71–88.

[ref5] Bandura A. (1997). Self-efficacy: The Exercise of Control. New York: W. H. Freeman.

[ref6] Bass B. M. (1985). Leadership and Performance beyond Expectations. New York: Free Press

[ref7] Bass B. M. Avolio B. J. (1994). Improving Organizational Effectiveness through Transformational Leadership. Thousand Oaks, CA SAGE Publication.

[ref8] Bass B. M. Stogdill R. M. (1990). Bass and Stogdill’s Handbook of Leadership: Theory, Research, and Managerial Applications, 3rd Edn New York: Free Press.

[ref9] Boehnlein P. Baum M. (2022). Does job crafting always lead to employee well-being and performance? Meta-analytical evidence on the moderating role of societal culture. Int. J. Hum. Resour. Manag. 33, 647–685. doi: 10.1080/09585192.2020.1737177

[ref10] Bothma F. C. Lloyd S. Khapova S. (2015). “Work Identity: Clarifying the Concept,” in Conceptualising and Measuring Work Identity. eds. Jansen P. G. W. Robert G. (Berlin: Springer), 23–51.

[ref11] Bunderson J. S. Thompson J. A. (2009). The call of the wild: zookeepers, callings, and the double-edged sword of deeply meaningful work. Adm. Sci. Q. 54, 32–57. doi: 10.2189/asqu.2009.54.1.32

[ref12] Cai W. El Baroudi S. Khapova S. N. Xu B. Kraimer M. L. (2021). Career calling and team member proactivity: the roles of living out a calling and mentoring. Appl. Psychol. 1– 25, 71, 587–611. doi: 10.1111/apps.12340

[ref13] Carless S. A. Wearing A. J. Mann L. (2000). A short measure of transformational leadership. J. Bus. Psychol. 14, 389–405. doi: 10.1023/A:1022991115523

[ref14] Cha E.-S. Kim K. H. Erlen J. A. (2007). Translation of scales in cross-cultural research: issues and techniques. J. Adv. Nurs. 58, 386–395. doi: 10.1111/j.1365-2648.2007.04242.x 17442038

[ref15] Chang P. C. Rui H. Lee A. Y. (2020). How having a calling leads to job crafting: a moderated mediation model. Front. Psychol. 11:552828. doi: 10.3389/fpsyg.2020.552828, PMID: 33041919 PMC7522331

[ref16] Chen Z. Zhu J. Zhou M. (2015). How does a servant leader fuel the service fire? A multilevel model of servant leadership, individual self identity, group competition climate, and customer service performance. J. Appl. Psychol. 100, 511–521. doi: 10.1037/a0038036, PMID: 25314366

[ref17] Cheng S. Q. Costantini A. Zhou H. Wang H. J. (2022). A self-enhancement perspective on organizational socialization: newcomer core self-evaluations, job crafting, and the role of leaders’ developmental coaching. Eur. J. Work Organ. Psy. 31, 908–921. doi: 10.1080/1359432X.2022.2077724

[ref18] Chiesa R. Van der Heijden B. I. Mazzetti G. Mariani M. G. Guglielmi D. (2020). “It is all in the game!”: the role of political skill for perceived employability enhancement. J. Career Dev. 47, 394–407. doi: 10.1177/0894845319832666

[ref19] Conway J. M. Lance C. E. (2010). What reviewers should expect from authors regarding common method bias in organizational research. J. Bus. Psychol. 25, 325–334. doi: 10.1007/s10869-010-9181-6

[ref20] Cooper B. Eva N. Fazlelahi F. Z. Newman A. Lee A. Obschonka M. (2020). Addressing common method variance and endogeneity in vocational behavior research: a review of the literature and suggestions for future research. J. Vocat. Behav. 121:103472. doi: 10.1016/j.jvb.2020.103472

[ref21] Costantini A. Demerouti E. Ceschi A. Sartori R. (2022). Implementing job crafting behaviors: exploring the effects of a job crafting intervention based on the theory of planned behavior. J. Appl. Behav. Sci. 58, 477–512. doi: 10.1177/002188632097591

[ref22] Crant J. M. (2000). Proactive behavior in organizations. J. Manag. 26, 435–462. doi: 10.1177/014920630002600304

[ref23] De Vries R. E. Roe R. A. Taillieu T. C. (2002). Need for leadership as a moderator of the relationships between leadership and individual outcomes. Leadersh. Q. 13, 121–137. doi: 10.1016/S1048-9843(02)00097-8

[ref24] Del Boca F. K. Noll J. A. (2000). Truth or consequences: the validity of self-report data in health services research on addictions. Addiction 95, 347–360. doi: 10.1046/j.1360-0443.95.11s3.5.x 11132362

[ref25] Den Hartog D. N. Belschak F. D. (2012). When does transformational leadership enhance employee proactive behavior? The role of autonomy and role breadth self-efficacy. J. Appl. Psychol. 97, 194–202. doi: 10.1037/a0024903, PMID: 21842977

[ref26] Dik B. J. Duffy R. D. Eldridge B. M. (2009). Calling and vocation in career counseling: recommendations for promoting meaningful work. Prof. Psychol. Res. Pract. 40, 625–632. doi: 10.1037/a0015547

[ref27] Dik B. J. Eldridge B. M. Steger M. F. Duffy R. D. (2012). Development and validation of the calling and vocation questionnaire (CVQ) and brief calling scale (BCS). J. Career Assess. 20, 242–263. doi: 10.1177/1069072711434410

[ref28] Duffy R. D. Autin K. L. (2013). Disentangling the link between perceiving a calling and living a calling. J. Couns. Psychol. 60, 219–227. doi: 10.1037/a0031934, PMID: 23438413

[ref29] Duffy R. D. Dik B. J. Douglass R. P. England J. W. Velez B. L. (2018). Work as a calling: a theoretical model. J. Couns. Psychol. 65, 423–439. doi: 10.1037/cou0000276 29999369

[ref30] Duffy R. D. Dik B. J. Steger M. F. (2011). Calling and work-related outcomes: career commitment as a mediator. J. Vocat. Behav. 78, 210–218. doi: 10.1016/j.jvb.2010.09.013

[ref31] Fiedler F. E. (1967). A Theory of Leadership Effectiveness. New York: McGraw-Hill

[ref32] Frederick D. E. VanderWeele T. J. (2020). Longitudinal meta-analysis of job crafting shows positive association with work engagement. Cogent. Psychol. 7, 1–19. doi: 10.1080/23311908.2020.1746733

[ref33] Golden-Biddle K. Rao H. (1997). Breaches in the boardroom: organizational identity and conflicts of commitment in a nonprofit organization. Organ. Sci. 8, 593–611. doi: 10.1287/orsc.8.6.593

[ref34] Griffin M. A. Parker S. K. Mason C. M. (2010). Leader vision and the development of adaptive and proactive performance: a longitudinal study. J. Appl. Psychol. 95, 174–182. doi: 10.1037/a0017263, PMID: 20085414

[ref35] Hall D. T. Chandler D. E. (2005). Psychological success: when the career is a calling. J. Organ. Behav. 26, 155–176. doi: 10.1002/job.301

[ref36] Harzer C. Ruch W. (2016). Your strengths are calling: preliminary results of a web-based strengths intervention to increase calling. J. Happiness Stud. 17, 2237–2256. doi: 10.1007/s10902-015-9692-y

[ref37] Hayes A. F. (2013). PROCESS SPSS Macro [Computer software and manual].

[ref38] Hayes A. F. (2015). An index and test of linear moderated mediation. Multivar. Behav. Res. 50, 1–22. doi: 10.1080/00273171.2014.962683 26609740

[ref39] Hersey P. Blanchard K. H. (1984). The Management of Organizational Behavior, 4th Edn Englewood Cliffs, NJ: Prentice Hall.

[ref40] Hetland J. Hetland H. Bakker A. B. Demerouti E. (2018). Daily transformational leadership and employee job crafting: the role of promotion focus. Eur. Manag. J. 36, 746–756. doi: 10.1016/j.emj.2018.01.002

[ref41] House R. J. (1971). A path-goal theory of leader effectiveness. Adm. Sci. Q. 16, 321–339. doi: 10.2307/2391905

[ref42] Hu L. T. Bentler P. M. (1999). Cutoff criteria for fit indexes in covariance structure analysis: conventional criteria versus new alternatives. Struct. Equ. Model. Multidiscip. J. 6, 1–55. doi: 10.1080/10705519909540118

[ref43] Hui C. H. Triandis H. C. (1985). Measurement in cross-cultural psychology: a review and comparison of strategies. J. Cross Cult. Psychol. 16, 131–152. doi: 10.1177/0022002185016002001

[ref44] Ji Y. Yoon H. J. (2021). The effect of servant leadership on self-efficacy and innovative behaviour: verification of the moderated mediating effect of vocational calling. Administr. Sci. 11:39. doi: 10.3390/admsci11020039

[ref45] Jones G. R. (1986). Socialization tactics, self-efficacy, and newcomers' adjustments to organizations. Acad. Manag. J. 29, 262–279. doi: 10.2307/256188

[ref46] Kelloway E. K. Barling J. Helleur J. (2000). Enhancing transformational leadership: the roles of training and feedback. Leader. Organ. Develop. J. 21, 145–149. doi: 10.1108/01437730010325022

[ref47] Kroger J. (1997). Gender and identity: the intersection of structure, content, and context. Sex Roles 36, 747–770. doi: 10.1023/A:1025627206676

[ref48] Kundu A. Ghose A. (2016). The relationship between attitude and self efficacy in mathematics among higher secondary students. J. Humanit. Soc. Sci. 21, 25–31. doi: 10.9790/0837-2104052531

[ref49] Li N. Chiaburu D. S. Kirkman B. L. Xie Z. (2013). Spotlight on the followers: An examination of moderators of relationships between transformational leadership and subordinates’ citizenship and taking charge. Pers. Psychol. 66, 225–260. doi: 10.1111/peps.12014

[ref50] Lim M. Ha Y. J. Oh D. J. Sohn Y. W. (2014). Validation of the Korean version of job crafting questionnaire (JCQ-K). Korean Corporat. Manag. Rev. 21, 181–206.

[ref51] Masdonati J. Massoudi K. Blustein D. L. Duffy R. D. (2022). Moving toward decent work: application of the psychology of working theory to the school-to-work transition. J. Career Dev. 49, 41–59. doi: 10.1177/0894845321991681

[ref52] Mazzetti G. Paolucci A. Guglielmi D. Vannini I. (2020). The impact of learning strategies and future orientation on academic success: the moderating role of academic self-efficacy among italian undergraduate students. Educat. Sci. 10:134. doi: 10.3390/educsci10050134

[ref53] Miraglia M. Cenciotti R. Alessandri G. Borgogni L. (2017). Translating self-efficacy in job performance over time: the role of job crafting. Hum. Perform. 30, 254–271. doi: 10.1080/08959285.2017.1373115

[ref54] Muller D. Judd C. M. Yzerbyt V. Y. (2005). When moderation is mediated and mediation is moderated. J. Pers. Soc. Psychol. 89, 852–863. doi: 10.1037/0022-3514.89.6.852 16393020

[ref55] Naeem R. M. Channa K. A. Hameed Z. Ali Arain G. Islam Z. U. (2021). The future of your job represents your future: a moderated mediation model of transformational leadership and job crafting. Pers. Rev. 50, 207–224. doi: 10.1108/PR-07-2019-0404

[ref56] Ng T. W. H. Feldman D. C. (2007). The school-to-work transition: a role identity perspective. J. Vocat. Behav. 71, 114–134. doi: 10.1016/j.jvb.2007.04.004

[ref57] Nielsen K. Yarker J. Randall R. Munir F. (2009). The mediating effects of team and self-efficacy on the relationship between transformational leadership, and job satisfaction and psychological well-being in healthcare professionals: a cross-sectional questionnaire survey. Int. J. Nurs. Stud. 46, 1236–1244. doi: 10.1016/j.ijnurstu.2009.03.001, PMID: 19345946

[ref58] Park J. Sohn Y. W. Ha Y. J. (2016). South Korean salespersons’ calling, job performance, and organizational citizenship behavior: the mediating role of occupational self-efficacy. J. Career Assess. 24, 415–428. doi: 10.1177/1069072715599354

[ref59] Parker S. K. Williams H. M. Turner N. (2006). Modeling the antecedents of proactive behavior at work. J. Appl. Psychol. 91, 636–652. doi: 10.1037/0021-9010.91.3.636, PMID: 16737360

[ref60] Petrou P. Demerouti E. Schaufeli W. B. (2015). Job crafting in changing organizations: antecedents and implications for exhaustion and performance. J. Occup. Health Psychol. 20, 470–480. doi: 10.1037/a0039003, PMID: 25798717

[ref61] Pijpker R. Kerksieck P. Tušl M. De Bloom J. Brauchli R. Bauer G. F. (2022). The role of off-job crafting in burnout prevention during COVID-19 crisis: a longitudinal study. Int. J. Environ. Res. Public Health 19:2146. doi: 10.3390/ijerph19042146, PMID: 35206330 PMC8872592

[ref62] Podsakoff P. M. Mac Kenzie S. B. Podsakoff N. P. (2012). Sources of method bias in social science research and recommendations on how to control it. Annu. Rev. Psychol. 63, 539–569. doi: 10.1146/annurev-psych-120710-100452 21838546

[ref63] Rigotti T. Schyns B. Mohr G. (2008). A short version of the occupational self-efficacy scale: structural and construct validity across five countries. J. Career Assess. 16, 238–255. doi: 10.1177/1069072707305763

[ref64] Rudolph C. W. Katz I. M. Lavigne K. N. Zacher H. (2017). Job crafting: a meta-analysis of relationships with individual differences, job characteristics, and work outcomes. J. Vocat. Behav. 102, 112–138. doi: 10.1016/j.jvb.2017.05.008

[ref65] Saks A. M. Gruman J. A. Cooper-Thomas H. (2011). The neglected role of proactive behavior and outcomes in newcomer socialization. J. Vocat. Behav. 79, 36–46. doi: 10.1016/j.jvb.2010.12.007

[ref66] Sakuraya A. Shimazu A. Imamura K. Namba K. Kawakami N. (2016). Effects of a job crafting intervention program on work engagement among Japanese employees: a pretest-posttest study. BMC Psychol. 4, 1–9. doi: 10.1186/s40359-016-0157-9, PMID: 27776553 PMC5078879

[ref67] Schoon I. Heckhausen J. (2019). Conceptualizing individual agency in the transition from school to work: a social-ecological developmental perspective. Adolesc. Res. Rev. 4, 135–148. doi: 10.1007/s40894-019-00111-3

[ref68] Schwartz S. J. Luyckx K. Vignoles V. L. (2011). Handbook of Identity Theory and Research. New York: Springer.

[ref69] Shepherd D. A. Williams T. A. (2018). Hitting rock bottom after job loss: bouncing back to create a new positive work identity. Acad. Manag. Rev. 43, 28–49. doi: 10.5465/amr.2015.0102

[ref70] Shim Y. Yoo S. (2012). Development and validation of the Korean version of the calling and vocation questionnaire (CVQ-K). Korean J. Couns. Psychother. 24, 847–872.

[ref71] Shrout P. E. Bolger N. (2002). Mediation in experimental and nonexperimental studies: new procedures and recommendations. Psychol. Methods 7, 422–445. doi: 10.1037/1082-989X.7.4.422, PMID: 12530702

[ref72] Siemsen E. Roth A. Oliveira P. (2010). Common method bias in regression models with linear, quadratic, and interaction effects. Organ. Res. Methods 13, 456–476. doi: 10.1177/109442810935124

[ref73] Slemp G. R. Kern M. L. Vella-Brodrick D. A. (2015). Workplace well-being: the role of job crafting and autonomy support. Psychol. Well Being 5, 1–17. doi: 10.1186/s13612-015-0034-y

[ref74] Slemp G. R. Vella-Brodrick D. A. (2013). The job crafting questionnaire: a new scale to measure the extent to which employees engage in job crafting. Int. J. Wellbeing 3, 126–146. doi: 10.5502/ijw.v3i2.1

[ref75] Stets J. E. Burke P. J. (2000). Identity theory and social identity theory. Soc. Psychol. Q. 63, 224–237. doi: 10.2307/2695870

[ref76] Stock G. Banks G. C. Voss E. N. Tonidandel S. Woznyj H. (2022). Putting leader (follower) behavior back into transformational leadership: a theoretical and empirical course correction. Leadersh. Q.:101632. doi: 10.1016/j.leaqua.2022.101632

[ref77] Sung S. C. Huang H. C. Lin M. H. (2015). Relationship between the knowledge, attitude, and self-efficacy on sexual health care for nursing students. J. Prof. Nurs. 31, 254–261. doi: 10.1016/j.profnurs.2014.11.001, PMID: 25999199

[ref78] Super D. E. (1990). “A life-span, life-space approach to career development” in Career Choice and Development: Applying Contemporary Theories to Practice, 2nd Edn. eds. Brown D. Brooks L. (San Francisco, CA: Jossey-Bass), 197–261.

[ref79] Taris T. W. Kessler S. R. Kelloway E. K. (2021). Strategies addressing the limitations of cross-sectional designs in occupational health psychology: what they are good for (and what not). Work Stress 35, 1–5. doi: 10.1080/02678373.2021.1888561

[ref80] Tims M. Bakker A. B. (2010). Job crafting: towards a new model of individual job redesign. SA J. Ind. Psychol. 36, 1–9. doi: 10.4102/sajip.v36i2.841

[ref81] Tims M. Bakker A. B. Derks D. (2013). The impact of job crafting on job demands, job resources, and well-being. J. Occup. Health Psychol. 18, 230–240. doi: 10.1037/a0032141, PMID: 23506549

[ref82] Tims M. Bakker A. B. Derks D. (2014). Daily job crafting and the self-efficacy – performance relationship. J. Manag. Psychol. 29, 490–507. doi: 10.1108/JMP-05-2012-0148

[ref83] Tims M. Bakker A. B. Derks D. (2015). Job crafting and job performance: a longitudinal study. Eur. J. Work Organ. Psy. 24, 914–928. doi: 10.1080/1359432X.2014.969245

[ref84] Tims M. Derks D. Bakker A. B. (2016). Job crafting and its relationships with person-job fit and meaningfulness: a three-wave study. J. Vocat. Behav. 92, 44–53. doi: 10.1016/j.jvb.2015.11.007

[ref85] Tims M. Twemlow M. Fong C. Y. M. (2021). A state-of-the-art overview of job-crafting research: current trends and future research directions. Career Dev. Int. 27, 54–78. doi: 10.1108/CDI-08-2021-0216

[ref86] van den Heuvel M. Demerouti E. Peeters M. C. W. (2015). The job crafting intervention: effects on job resources, self-efficacy, and affective well-being. J. Occup. Organ. Psychol. 88, 511–532. doi: 10.1111/joop.12128

[ref88] van Wingerden J. Bakker A. B. Derks D. (2017). The longitudinal impact of a job crafting intervention. Eur. J. Work Organ. Psy. 26, 107–119. doi: 10.1080/1359432X.2016.1224233

[ref89] Verelst L. De Cooman R. Verbruggen M. van Laar C. Meeussen L. (2021). The development and validation of an electronic job crafting intervention: testing the links with job crafting and person-job fit. J. Occup. Organ. Psychol. 94, 338–373. doi: 10.1111/joop.12351

[ref90] Vroom V. H. (1982). Work and Motivation. Malabar, FL: R. E. Krieger Publishing Company.

[ref91] Wang H. J. Chen X. Lu C. Q. (2020). When career dissatisfaction leads to employee job crafting: the role of job social support and occupational self-efficacy. Career Dev. Int. 25, 337–354. doi: 10.1108/CDI-03-2019-0069

[ref92] Wang H. J. Demerouti E. Bakker A. B. (2016). “A review of job crafting research: the role of leader behaviors in cultivating successful job crafters,” in Proactivity at Work (Series in Organization and Management). eds. Parker S. K. Bindl U. K. (Oxfordshire: Routledge), 95–122.

[ref93] Wang H. J. Demerouti E. Le Blanc P. (2017). Transformational leadership, adaptability, and job crafting: the moderating role of organizational identification. J. Vocat. Behav. 100, 185–195. doi: 10.1016/j.jvb.2017.03.009

[ref94] Wang H. Wang X. Li J. (2018). Is new generation employees’ job crafting beneficial or detrimental to organizations in China? Participative decision-making as a moderator. Asia Pac. Bus. Rev. 24, 543–560. doi: 10.1080/13602381.2018.1451129

[ref95] Westland J. C. (2010). Lower bounds on sample size in structural equation modeling. Electron. Commer. Res. Appl. 9, 476–487. doi: 10.1016/j.elerap.2010.07.003

[ref96] Weston R. Gore P. A. (2006). A brief guide to structural equation modeling. Couns. Psychol. 34, 719–751. doi: 10.1177/0011000006286345

[ref97] Wrzesniewski A. Dutton J. E. (2001). Crafting a job: Revisioning employees as active crafters of their work. Acad. Manag. Rev. 26, 179–201. doi: 10.5465/amr.2001.4378011

[ref98] Wrzesniewski A. McCauley C. Rozin P. Schwartz B. (1997). Jobs, careers, and callings: People’s relations to their work. J. Res. Pers. 31, 21–33. doi: 10.1006/jrpe.1997.2162

[ref99] Yeşilkaya M. Yıldız T. (2022). What do expectations change? Optimistic expectations, job crafting, job satisfaction and a new theoretical model. Int. J. Organ. Anal. Adv. Publicat. doi: 10.1108/IJOA-01-2022-3111

[ref100] Yukl G. (1999). An evaluation of conceptual weaknesses in transformational and charismatic leadership theories. Leadersh. Q. 10, 285–305. doi: 10.1016/S1048-9843(99)00013-2

[ref101] Zhang C. Hirschi A. You X. (2021). Trajectories of calling in the transition from university to work: a growth mixture analysis. J. Career Assess. 29, 98–114. doi: 10.1177/1069072720931010

[ref102] Zhang F. Parker S. K. (2019). Reorienting job crafting research: a hierarchical structure of job crafting concepts and integrative review. J. Organ. Behav. 40, 126–146. doi: 10.1002/job.2332

