# Supplementary material for: Encouraging job crafting in the workplace for newcomers: A two-year multi-wave study
Source: Front Psychol. 2022 Dec 9;13:1003276. doi: 10.3389/fpsyg.2022.1003276 (PMC9784467; doi:10.3389/fpsyg.2022.1003276)
Supplement: Supplementary file 1 [file Data_Sheet_1.docx]

**Appendix**

**Figure 1**

*The Moderated Mediation Model with Standardized Coefficients for Task Crafting*


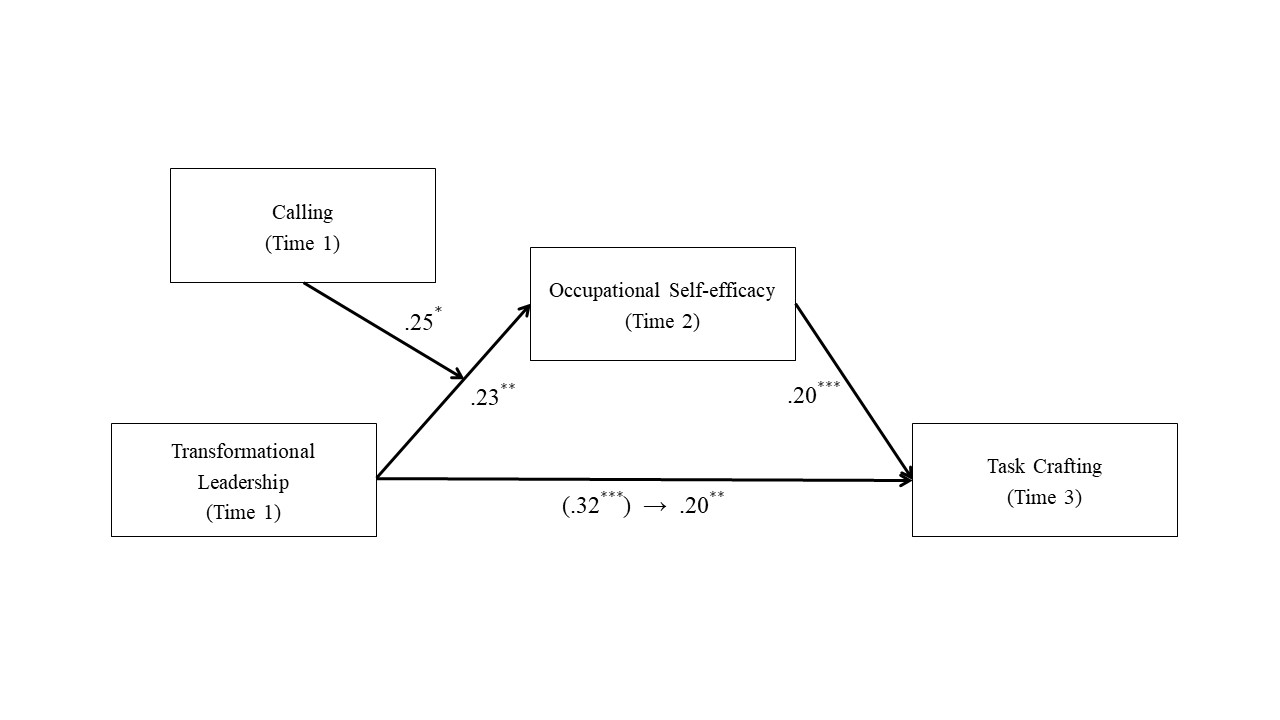


***Note***. The number in parentheses is the direct path coefficient before the other variables are entered. ^*^*p* < .05, ^**^*p* < .01, ^***^*p* < .001.

**Figure 2**

*The Moderated Mediation Model with Standardized Coefficients for Cognitive Crafting*


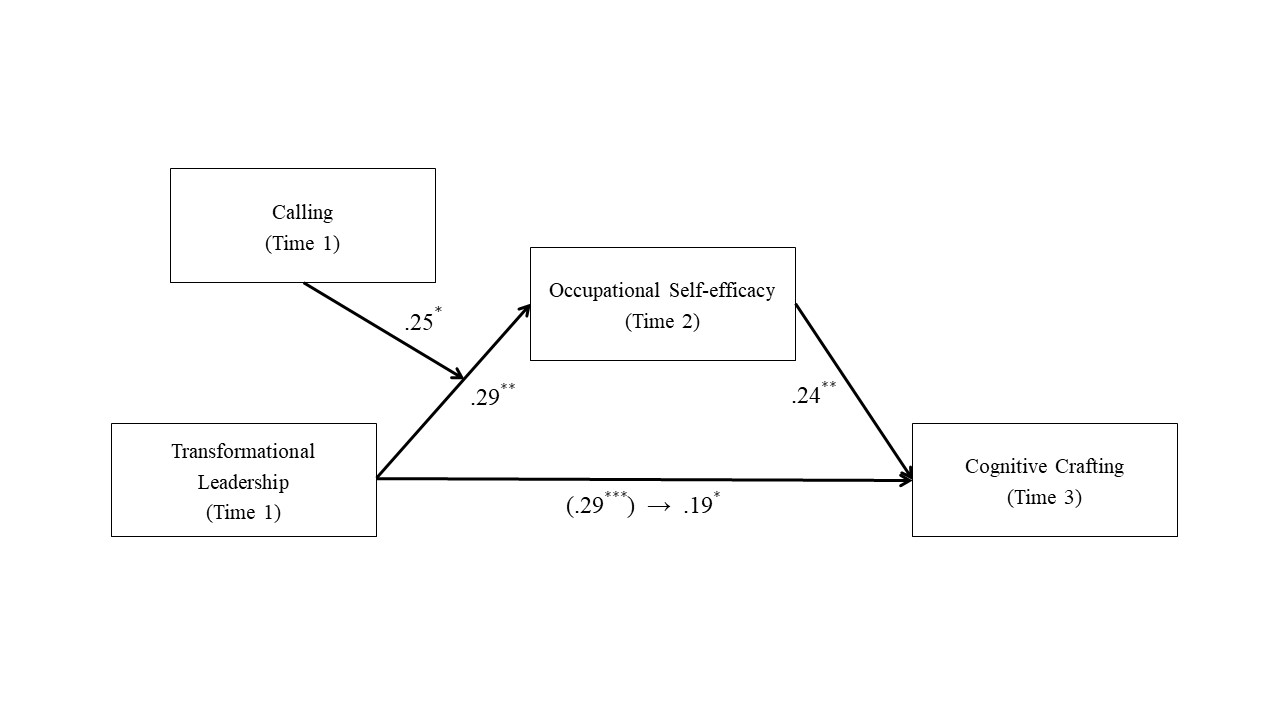


***Note***. The number in parentheses is the direct path coefficient before the other variables are entered. ^*^*p* < .05, ^**^*p* < .01, ^***^*p* < .001.

**Figure 3**

*The Moderated Mediation Model with Standardized Coefficients for Relational Crafting*


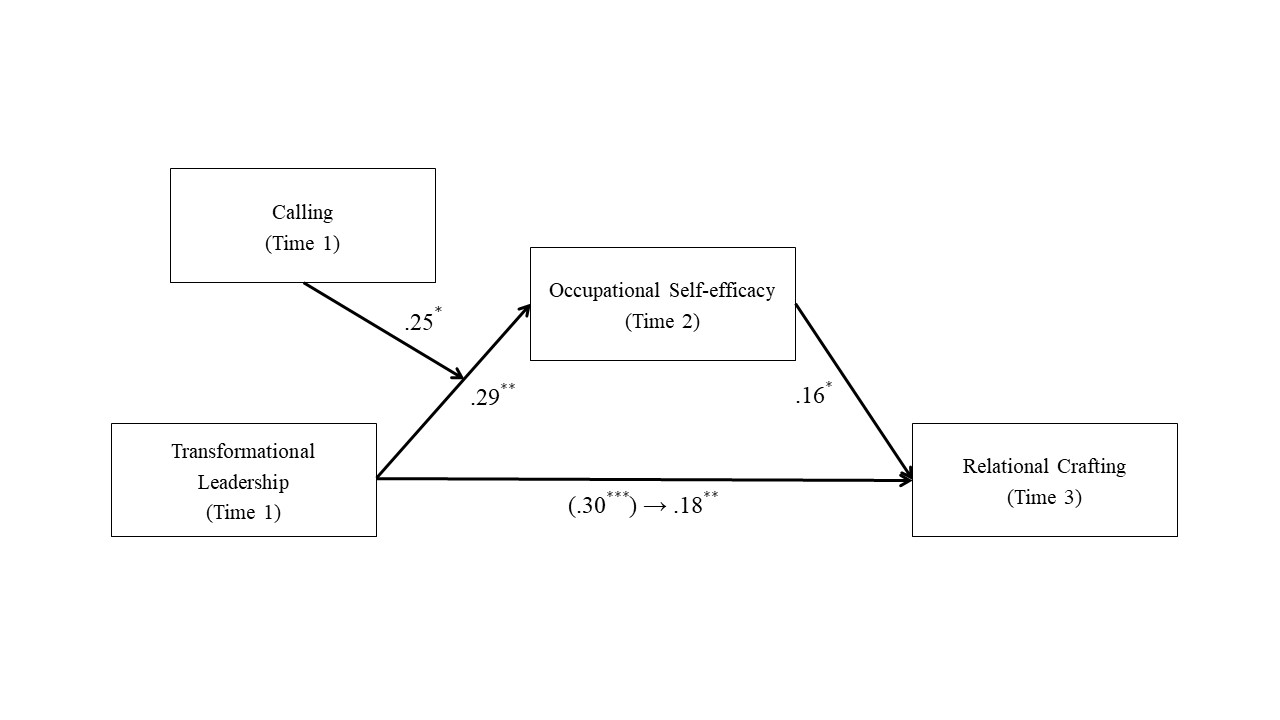


***Note***. The number in parentheses is the direct path coefficient before the other variables are entered. ^*^*p* < .05, ^**^*p* < .01, ^***^*p* < .001.
